# Pregnancy and Motherhood Experiences of Turkish Women Anaesthesiologists in Training and Work Life

**DOI:** 10.4274/TJAR.2026.252337

**Published:** 2026-04-15

**Authors:** Aslıhan Güleç Kılıç, Beyza Büyükgebiz Yeşil, Selin Erel

**Affiliations:** 1 Göksun State Hospital, Clinic of Anaesthesiology and Reanimation, Kahramanmaraş, Türkiye; 2 Kahramanmaraş State Hospital, Clinic Anaesthesiology and Reanimation, Kahramanmaraş, Türkiye; 3 Gazi University Faculty of Medicine, Department of Anaesthesiology and Reanimation, Ankara, Türkiye

**Keywords:** Anaesthesiology, breast feeding, pregnancy, survey

## Abstract

**Objective:**

To evaluate the experiences and perceived obstacles related to pregnancy and motherhood among Turkish women anaesthesiologists and to identify work-related factors associated with considering resignation during pregnancy.

**Methods:**

This cross-sectional survey targeted female anaesthesiologists working in or trained in Türkiye. Participants responded to 39 items across five domains: (1) demographics and professional background, (2) fertility intentions and childbearing decisions, (3) pregnancy history and obstetric outcomes, (4) impact of pregnancy on training, work-life, and academic productivity, and (5) breastfeeding practices and workplace support. Responses were collected over a two-week period without reminder emails and subsequently analyzed.

**Results:**

A total of 334 women anaesthesiologists completed the survey; 55.7% had at least one child and 36.8% were childless. Among those without children, 41.3% still desired motherhood, and working conditions were significantly associated with uncertainty or reluctance toward childbearing (*P* < 0.001). 39.8% of participants delayed antenatal visits due to workload. Most participants (84.2%) returned early from maternity leave, and 75.5% reported reduced academic productivity during pregnancy or motherhood. Among those who breastfed or pumped at work, 47.8% discontinued earlier than intended, largely because of work-related constraints. Overall, 35.2% considered resigning during pregnancy, and work-related disruption of antenatal care was the only independent predictor of intent to resign (odds ratio 0.28, 95% confidence interval 0.12-0.61, *P*=0.001).

**Conclusion:**

Turkish pregnant anaesthesiologists frequently experienced disruptions in antenatal care, shortened maternity leave, reduced academic productivity, and inadequate breastfeeding support. Working conditions significantly influence both fertility decisions and intentions to resign.

**Main Points**

• Pregnancy and motherhood are common among Turkish women anaesthesiologists, yet working conditions frequently disrupt antenatal care and shorten maternity leave.

• Departmental workloads, shift patterns, and cultural expectations strongly influence fertility planning, leading many individuals to delay or reconsider childbearing.

• Workplace breastfeeding support remains insufficient, with structural and cultural barriers leading to early discontinuation of breastfeeding.

• Disrupted access to antenatal care independently predicted an intent to resign, emphasising the need for institutional policies that safeguard maternal well-being and workforce retention.

## Introduction

The proportion of women in the Turkish and international medical workforce has increased steadily over the past decade.^1, 2^ While women now represent a substantial share of physicians and medical residents globally, the gender distribution in Türkiye presents a unique pattern. Although the total number of female physicians in Türkiye remains lower than that of male physicians, the field of anaesthesiology exhibits a notable female predominance.^3^

Despite the increasing representation of female anaesthesiologists over the years, women continue to encounter significant obstacles in their professional lives. These challenges include persistent traditional gender norms, the gender pay gap, and underrepresentation in academic leadership roles.^4, 5, 6, 7, 8^ Among these issues, the difficulties faced during pregnancy and early motherhood are particularly prominent. Studies conducted among female physicians indicate that societal expectations regarding childcare and the unequal distribution of domestic responsibilities continue to shape career experiences. These factors heavily influence the professional lives of women, particularly during pregnancy and the postpartum period.^4, 5^

These challenges may be amplified in anaesthesiology, a specialty characterised by demanding on-call schedules, night shifts, occupational exposure concerns, and a high degree of clinical responsibility — factors known to influence working conditions for pregnant clinicians.^9^ A nationwide survey of women anaesthesiologists conducted by the American Society of Anesthesiologists (ASA) found that work demands frequently led women to delay childbearing, alter desired family size, or modify career plans.^10^ Similarly, studies from Europe have documented substantial variation in institutional support for pregnant anaesthesiologists, inconsistent occupational adaptations, and persistent concerns regarding environmental exposures in the operating room.^11, 12^ In addition to these structural barriers, several studies describe a perceived “motherhood penalty” among female anaesthesiology residents. Program directors in the United States report concerns that pregnancy or parental leave negatively affects female residents’ academic progression and procedural experience, despite a lack of objective evidence supporting these perceptions.^13^ Other surveys identify difficulties related to breastfeeding arrangements, stigma surrounding maternity leave, and reduced access to mentorship for women who become mothers during training.^14, 15^

Despite growing international attention to this topic, data regarding pregnancy, motherhood, and career progression among women anaesthesiologists in Türkiye remain limited. Findings from American or European settings may not be directly generalizable to Türkiye given the country’s unique sociocultural context, specific maternity legislation, institutional structures, and significantly higher patient-to-doctor ratios. Therefore, understanding how Turkish women anaesthesiologists perceive pregnancy and motherhood is essential for shaping workplace policies, improving educational environments, and supporting career sustainability within the specialty.

The present study aims to evaluate the attitudes, perceived barriers, and lived experiences of Turkish women anaesthesiologists regarding pregnancy and motherhood during training and professional life.

## Methods

This study was designed as a national, cross-sectional, web-based survey to evaluate the attitudes, experiences, and perceived challenges related to pregnancy and motherhood among women anaesthesiologists in Türkiye. Ethical approval for the study was obtained from Gazi University Rectorate Ethics Commission (approval no.: 2025-1882, date: 11/11/2025). Participation was voluntary, anonymous, and self-administered. No financial incentives were offered to survey participants, and all participants provided electronic informed consent prior to initiating the survey. This study adheres to the Checklist for Reporting Results of Internet E-Surveys guidelines, which outline methodological and reporting standards for the design, conduct, and presentation of internet-based survey research.^16^

The questionnaire was developed by the authors based on the survey instrument used in a previous study.^14^ The authors subsequently modified it in line with their opinions and adapted it to the specific context of Turkish anaesthesiology training and work culture. The final instrument consisted of 39 items, structured into five domains: demographics and professional background; fertility intentions and childbearing decisions; pregnancy history and obstetric outcomes; impact of pregnancy on training, work-life, and academic productivity; and breastfeeding practices and workplace support (Supplement 1). The item formats included single-choice questions, multiple-choice questions, numerical entries, and five-point Likert scales scored from 1 (strongly disagree) to 5 (strongly agree).

The survey was created and hosted using Typeform® (Typeform S.L., Barcelona, Spain), a secure online survey platform. The survey link was distributed through multiple channels to ensure wide national reach via email lists of the Turkish Society of Anesthesiology and Reanimation (TSAR) and via peer-to-peer professional email addresses for a two-week period. Reminder emails were intentionally omitted to reduce the risk of survey fatigue and to ensure that participation remained entirely voluntary, without any perceived pressure. Participants were able to complete the survey once. To ensure data integrity, IP addresses were monitored to prevent duplicate entries and were subsequently deleted from the final dataset to maintain complete anonymity. With respect to participant selection and data quality, the study population was strictly limited to women anaesthesiologists, and male colleagues were excluded. Additionally, only fully completed questionnaires were included in the final analysis; incomplete submissions were excluded. All responses were stored on a secure and password-protected server accessible only to the study team.

### Statistical Analysis

Data are analysed using SPSS (version 26, IBM Corp., Armonk, NY). Categorical variables are summarised as frequencies and percentages. Continuous variables are presented as mean ± standard deviation or median (interquartile range), depending on distribution.

Comparisons between subgroups (e.g., residents vs specialists; mothers vs. non-mothers) are performed using χ^2^ or Fisher’s exact tests for categorical data, and independent-samples t-tests or Mann-Whitney U tests for continuous variables, as appropriate. Statistical significance is defined as *P* < 0.05.

Variables associated with intent to resign at the univariable level (*P* < 0.05) were entered into a multivariable logistic regression using a backward stepwise (likelihood-ratio) method. Resignation intent was modelled as a binary outcome. Predictors were sequentially removed based on their contribution to model fit, with odds ratios (ORs) and 95% confidence intervals (CIs) reported for variables retained in the final model. Model adequacy was assessed using the Omnibus test, the Hosmer-Lemeshow test, and Nagelkerke R^2^.

## Results

The responses of 334 women anaesthesiologists who completed the survey were included in the analysis. An additional 173 participants initiated the survey but did not complete it. Based on unpublished administrative data obtained directly from TSAR, the email list includes 5,206 female anaesthesiologists (residents and specialists), yielding an estimated response rate of 6.4%. However, as the survey was also disseminated through peer-to-peer contacts via personal emails and the access routes of individual respondents could not be identified, the true denominator remains uncertain. Therefore, this estimate should be interpreted as an approximate lower-bound response rate. Most respondents were between 31 and 40 years of age, were attending anaesthesiologists, and had completed their residency in state university hospitals. Demographic characteristics and details of the residency institutions are summarized in Table 1.

Among the 334 respondents, 10 (3.0%) they were currently pregnant, 186 (55.7%) they had at least one child, and 138 (41.3%) had no children. Among those without children, 41.3% indicated that they wished to have children, while the remainder were either unsure or did not plan to become mothers. Among all 334 respondents, 196 (58.7%) either did not wish to have (more) children or were unsure about future childbearing, regardless of whether they already had children; working and training conditions were significantly associated with their decision (Table 2). While 43.8% of those who did not want children reported that working conditions influenced their decision, this proportion increased sharply to 81.4% among those who were unsure.

When comparing respondents who had never been pregnant with those who had been pregnant at least once, a significant overall difference in pregnancy-related workplace perception scores was observed (*P* < 0.05; Table 3). Women with previous pregnancy experience more strongly agreed that they felt discouraged about becoming a mother. In contrast, never-pregnant respondents reported greater agreement with perceiving negative attitudes toward pregnancy during training or in the workplace, feeling an increased workload due to a colleague’s maternity leave, and supporting a reduction in seniority upon returning from maternity leave.

Of the 196 respondents who had ever been pregnant, 64.8% (n = 127) stated that they were pregnant or were mothers during residency training. Overall, 39.8% (n = 78) reported delaying their prenatal care visits due to work demands. When asked whether they had ended their maternity leave earlier than planned, 84.2% (n = 165) reported that they had; the most frequently selected reasons were financial difficulties (49.7%, n = 82), fear of retaliation from the department leadership or hospital administration (30.9%, n = 51), concern about falling behind in knowledge or skills (29.1%, n = 48), and peer pressure (22.4%, n = 37). Seventy-five percent (n = 148) of respondents who had ever been pregnant reported that their academic work performance declined during pregnancy and/or motherhood. The most commonly reported contributors to this decline in academic performance were tiredness or sleep deprivation (77.0%, n = 114), difficulty balancing work and family responsibilities (62.2%, n = 92), insufficient workplace support (51.4%, n = 76), emotional distress (50.7%, n = 75), and, less frequently, discrimination (19.6%, n = 29).

Among the 136 participants who breastfed or pumped during working hours, 24.3% (n = 33) reported inadequate support from peers or faculty, and 39.7% (n = 54) experienced guilt during pumping breaks. A total of 47.8% (n = 65) discontinued lactation earlier than intended; of these, 82.9% attributed this to work-related factors. Additionally, 68.4% (n = 93) reported that an appropriate pumping room was either unavailable or inaccessible during their regular work schedule.

Overall, 35.2% (n = 69) reported having considered resigning during pregnancy. Chi-square analyses were conducted to explore the association between individual variables and resignation intent during pregnancy. Several factors were significantly associated with consideration of resignation. These included delays in antenatal follow-up due to workload (*P* < 0.001), pregnancy-related symptoms, such as gastrointestinal disturbance, backache, or memory difficulties (*P*=0.003), and perceived decline in academic work performance (*P*=0.04). To further evaluate the independent contributions of these variables, a backward-stepwise logistic regression was performed (Table 4). After sequential removal of non-contributing predictors, the final model retained two variables and remained significant overall. Work-related disruption of antenatal care emerged as the only statistically significant independent predictor of resignation intent (OR: 0.275, 95% CI: 0.124-0.608, *P*=0.001). Anaesthesiologists who experienced work-related disruption of antenatal care were significantly more likely to consider resigning than those without such disruption (OR: 0.28, 95% CI: 0.12-0.61). Pregnancy-related symptoms showed a not statistically significant trend toward an association with the outcome (OR: 0.394, *P*=0.07) and remained in the model based on likelihood-ratio criteria, though it did not reach statistical significance in the multivariable model. The final model demonstrated excellent calibration (Hosmer-Lemeshow *P*=0.95) and explained approximately 18 percent of the variance in resignation intent (Nagelkerke R^2^ = 0.18) (Table 4).

## Discussion

In this first national survey of Turkish women anaesthesiologists, pregnancy and motherhood were common during residency and were frequently accompanied by delayed antenatal care, shortened maternity leave, reduced academic productivity, and substantial barriers to breastfeeding at work. Working and training conditions strongly shaped fertility intentions and workplace perceptions; many participants described workload, departmental culture, and informal expectations as discouraging childbearing. Never-pregnant respondents were more likely to report feeling burdened by colleagues’ maternity leave, suggesting that the absence of structured coverage mechanisms may contribute to tension and stigma. This finding that disruption to antenatal care was the strongest factor associated with considering resignation underscores that current work patterns remain misaligned with basic perinatal needs.

Consistent with global trends, motherhood during anaesthesiology training was highly prevalent in our cohort.^10, 14, 16^ Almost two-thirds of respondents were pregnant or were mothers during residency. Considering reports that infertility rates may be higher among anaesthesiologists than in the general population, this finding is encouraging.^17^ On the other hand, it is concerning that working conditions and workplace culture played a major role in the decision of women without children not to pursue motherhood. Among those who were unsure, more than 80% identified workload and training demands as contributing factors; even among those who did not want children, nearly half cited working conditions. These findings suggest that the current structure of anaesthesiology training and service in Türkiye acts as a deterrent to childbearing for some women, rather than serving as a neutral background factor. Occupational hazards, such as exposure to waste anesthetic gases and radiation, and reports that female anaesthesiologists may be subjected to mobbing more frequently than their male colleagues, could also contribute to these findings.^17, 18, 19^Although these factors were not directly assessed in our survey, they provide plausible contextual considerations that may help interpret our findings and should be explored in future studies.

Beyond these well-recognized occupational risks and the traditional forms of workplace hostility encountered in surgical specialties, our findings point to a strained workplace climate regarding pregnancy. Women who had experienced pregnancy felt more discouraged from becoming mothers while in training or at work, whereas never-pregnant anaesthesiologists more often agreed that they bore an unfair share of the workload during colleagues’ maternity leave and were more likely to support reducing seniority after maternity leave. This pattern suggests that in the absence of clear staffing policies and formal coverage arrangements, pregnancy-related workload may be perceived as a zero-sum issue at the departmental level. Similar concerns about stigma, resentment over workload redistribution, and a perceived motherhood penalty have been described in surveys of anaesthesiologists and residents from North America and Europe, in which pregnancy is frequently regarded as disruptive both to the individual’s career and to colleagues covering clinical duties.^9, 10, 11, 12, 13, 14^ The divergent perceptions between never-pregnant and ever-pregnant respondents highlight a potentially self-perpetuating cycle. In the absence of clear, institutionally mandated coverage structures, colleagues who repeatedly cover additional calls or shifts for pregnant peers may come to view pregnancy as a private benefit with collective costs. This can increase stigma and make colleagues more supportive of penalising practices, such as reducing seniority following maternity leave. As a result, residents may be less willing to disclose a pregnancy early or to take their full entitlement to leave. Breaking this cycle will require making coverage arrangements a departmental responsibility rather than an informal negotiation among residents.

Among women who became pregnant or who were mothers during residency, many reported that basic perinatal needs were compromised by work. Although women physicians in Türkiye who are covered by the Civil Servants Law No. 657 have strong statutory protections, these provisions may not translate into full real-world use within anaesthesiology. Under Legislation No. 657, maternity leave totals 16 weeks (prenatal plus postnatal), and breastfeeding or lactation time is allocated.^20^ In addition, pregnant civil servants from 24 weeks’ gestation and those up to two years postpartum cannot be assigned night duty or night shifts. By comparison, at the European Union level, the legal baseline is at least 14 consecutive weeks of maternity-leave, whereas in the United States there is no universal federal paid maternity-leave entitlement for physicians, and the duration of paid leave often depends on employer policy; however, ACGME-accredited training programs must provide a minimum of 6 weeks of approved paid leave.^21, 22^ Despite these legal safeguards in Türkiye, the workflow of anaesthesiology, where uninterrupted patient monitoring and staffing continuity are essential, can make it difficult for physicians to consistently take leave, obtain shift exemptions, or access protected break entitlements. In our study, almost 40% of participants reported difficulty attending antenatal visits and 85% ended their maternity leave early. These proportions are similar to findings reported in countries with very different legal frameworks. Almost 40% reported delaying antenatal visits because of workload, a rate consistent with international surveys.^14^ These challenges pose a significant problem for pregnant physicians. In our multivariable analysis, work-related disruption of antenatal care was the most significant independent predictor of considering resignation from work during pregnancy. Anaesthesiologists who were unable to attend antenatal appointments because of work demands were more likely to report an intent to resign. Similar findings from European studies, in which pregnancy-related working conditions have led anaesthesiologists to consider leaving training programs, further support this interpretation.^11^ From a workforce perspective, ensuring protected time for antenatal care may therefore be a simple but important strategy to reduce avoidable stress and potential attrition among early-career anaesthesiologists.

Maternity leave is a key component of postpartum recovery and mental health.^23, 24^ Prior qualitative research has shown that an early return to work can heighten stress related to childcare, breastfeeding, and sleep deprivation.^25^ Although the specific reasons differ across countries with varying legal and cultural environments, such as rigid schedules, limited or unpaid maternity leave, and the absence of structured return-to-work support, the high rates of early termination of maternity leave in our cohort suggest that the pregnancy experiences of anaesthesiologists cannot be fully safeguarded through legislation alone.^9,10,11,13,14,15^ Instead, these patterns indicate a broader professional culture in which pregnancy and early motherhood are treated as individual burdens to be absorbed by the mother, rather than predictable and recurrent situations that require explicit accommodation through paid leave, flexible scheduling, and formal protection from informal sanctions.

In the present study, 75% of participants reported a decline in academic productivity during pregnancy and motherhood, which warrants particular attention in the academic setting. In many Turkish training and university hospitals, criteria for academic advancement are heavily weighted toward conference presentations, publications, and uninterrupted clinical activity. Women who become mothers may therefore be structurally disadvantaged, even when their clinical performance remains strong. This self-reported decline is broadly aligned with existing data: a scoping review of pregnancy in physicians found that, although measured work productivity and academic metrics were generally independent of pregnancy, colleagues frequently perceived pregnant physicians as less productive and more burdensome to the team.^26^ In residency settings, almost half of female residents and of department heads explicitly describe pregnant colleagues as less productive and believe that pregnancy and motherhood plans affect selection and advancement decisions.^27^ Beyond medicine, longitudinal analyses of academic careers show that parenthood explains much of the gender productivity gap by lowering mothers’ short-term publication output, even though parents as a group remain at least as productive as non-parents over the long term.^28^ Together, these findings suggest that even modest, time-limited reductions in scholarly activity around pregnancy can be magnified within systems that reward continuous output, resulting in a disproportionate productivity penalty for women who have children. These perceived or actual dips in scholarly output are also likely to intersect with impostor phenomenon, which affects an estimated 22-60% of physicians and is more prevalent among women in unsupportive institutional cultures.^29^ Recent data in anaesthesiology suggest that impostor syndrome disproportionately affects female and junior anaesthesiologists; pregnancy-related disruptions in academic productivity may therefore reinforce self-doubt and perceived inadequacy among those already at highest risk.^30^

In our cohort, the gender of the department head during residency was not associated with more favourable pregnancy-related perceptions or experiences, including antenatal care disruption, early return from maternity leave, or breastfeeding support. These findings are similar to those from an ASA resident survey, in which the presence of a female programme director or department chair did not meaningfully change leave characteristics, breastfeeding experiences, or feelings of guilt related to pregnancy and parenting during training.^14^ These results suggest that the gender of leaders alone is not sufficient; departmental culture, institutional policies, and their implementation appear to be at least as important. Strengthening leadership commitment to supporting working parents, alongside efforts to improve gender representation in leadership, may therefore be a more realistic strategy to recruit and retain women in anaesthesiology.^31^

From a workforce and policy perspective, in light of the challenges reported by survey respondents, structural, rather than individual, solutions are needed for pregnancy-related challenges in anaesthesiology. Departments should establish formal, institutionally supported coverage mechanisms for pregnancy and maternity leave instead of relying on informal peer-based arrangements. Protected time for antenatal care, predictable scheduling adjustments, and access to breastfeeding facilities should be integrated into departmental workflows. Clearer enforcement of existing legal protections through departmental protocols and leadership accountability may help reduce stigma and support retention of early-career women anaesthesiologists.

### Study Limitations

This study has limitations. Its cross-sectional, self-reported design precludes causal inference and is vulnerable to recall and social desirability bias, particularly for events that occurred earlier in participants’ careers. The survey instrument used in this study was neither formally validated nor pilot-tested prior to dissemination. As the study was designed to be exploratory and descriptive, the questionnaire aimed to capture a broad snapshot of attitudes rather than function as a validated psychometric tool. While the items were developed based on a review of the relevant literature and aligned with the study objectives, the absence of formal validation may limit the precision and generalisability of the findings. Because the survey link was distributed via professional networks without a defined sampling frame, we could not calculate a true response rate. Similar to previous surveys, non-response bias cannot be excluded; it is possible that women with stronger experiences related to pregnancy or motherhood, whether positive or negative, were more likely to participate than those who felt less affected by these issues.^10, 32^ The sample was restricted to female anaesthesiologists, so the perspectives of male colleagues and departmental leadership on workload distribution, maternity leave, and breastfeeding policies were not directly assessed. Finally, we measured resignation intent rather than actual turnover and did not link responses to objective career outcomes; longitudinal studies would be required to determine whether the patterns observed here translate into concrete workforce losses.

## Conclusion

In this national survey of Turkish women anaesthesiologists, pregnancy and motherhood were common during residency and often accompanied by disrupted antenatal care, shortened maternity leave, perceived declines in academic productivity, and substantial barriers to breastfeeding at work. These findings indicate that pregnancy and early motherhood in anaesthesiology are still treated as individual challenges rather than as predictable workforce needs. The development and consistent implementation of explicit department-level policies, particularly those that ensure protected time for antenatal care, may be critical to retaining women in the specialty and supporting a sustainable, gender-inclusive anaesthesiology workforce in Türkiye.

## Ethics

**Ethics Committee Approval:** Ethical approval for the study was obtained from Gazi University Rectorate Ethics Commission (approval no.: 2025-1882, date: 11/11/2025).

**Informed Consent:** Participation was voluntary, anonymous, and self-administered. No financial incentives were offered to survey participants, and all participants provided electronic informed consent prior to initiating the survey.

## Supplementary Materials

Supplement 1 https://d2v96fxpocvxx.cloudfront.net/cf9d60d6-523c-458a-a2e6-78728d3ffbb0/documents/TJAR-2337_APPENDİX.docx

## Figures and Tables

**Table 1. Demographic Data of the Participants and Characteristic of Their Educational Instution:** 

**Demographics and characteristics**	**n (%), median (Q1-Q3)**
**Age groups of the participants (years), n (%)** • 24-30 • 31-40 • >40	- 75 (22.5) 137 (41.0) 122 (36.5)
**Residency/working status, n (%)** • Junior resident (≤2 years) • Senior resident (>2 years) • Attending anaesthesiologist • Consultant anaesthesiologists with academic title	- 35 (10.5) 85 (25.4) 176 (52.7) 38 (11.4)
**Institutional setting during residency training, n (%)** • State university hospital • Training and research hospital • City hospital • Private foundation university hospital	- - 196 (58.7) 109 (32.6) 25 (7.5) 4 (1.2)
**Co-residents during training, median (Q1-Q3)** • Average total resident number • Average total female resident number • Average percentage of female resident number	- - 30 (18-45) 17 (10-25) 60 (50-68.75)
**Academic faculty during training, median (Q1-Q3)** • Average total academic faculty number • Average total female academic faculty number • Average percentage of female faculty	- - 10 (5-14) 5 (2-8) 60 (40-71.43)
**Gender of the department head during training, n (%)** • Female • Male • Both	- - 140 (41.9) 142 (42.5) 52 (15.6)
**Presence of pregnant co-residents during training, n (%)**	306 (91.6)

n, number; Q, quartile

**Table 2. Effect of Working Conditions on Motherhood Intentions:** 

**Motherhood intention categories**	**Perceived impact of working conditions, n (%)**			**Total**	***P* value**
	**No**	**Yes**	**Partially**		
Uncertain	8 (18.6)	23 (52.49)	12 (52.17)	43	**<0.001**
Not planning	86 (56.21)	41 (26.8)	26 (16.99)	153	
Total	94	64	38	196	-

Chi-square test *P *< 0.05 was considered statistically significant

**Table 3. Pregnancy-Related Workplace Perception Scores by Previous Pregnancy Status:** 

**Statements**	**Never pregnant** **(n = 138)** **median (Q1-Q3)**	**Ever pregnant** **(n = 196)** **median (Q1-Q3)**	***P***
“I felt discouraged from becoming a mother during training/working life”	3 (2-4)	4 (2-5)	**0.014**
“I perceived negative attitudes toward pregnancy during my training/working life”	4 (2-5)	4 (3-5)	**0.004**
“I felt that I carried an unfair workload due to a colleague’s maternity leave”	2 (1-4)	2 (1-3)	**0.03**
“An employee returning from maternity leave should have her seniority reduced”	3 (1-4)	2 (1-3)	**0.026**

Mann-Whitney U test, *P *< 0.05 was considered statistically significant; Q, quartile

**Table 4. Multivariable Logistic Regression Analysis of Factors Associated with Resignation Intent During Pregnancy Among Anaesthesiologists:** 

-	**B**	**SE**	**Odds ratio (95% CI)**	***P***
Work related disruption of antenatal care	-1.29	0.41	0.28 (0.12-0.61)	**0.001**
Experiencing pregnancy associated symptoms	-0.93	0.52	0.39 (0.14-1.08)	0.07

Multivariable backward logistic regression analysis, *P *< 0.05 was considered statistically significant. Outcome variable was coded as 1= consideration of resignation and 0= no consideration. Independent variables were coded as 1= presence of exposure and 0= absence

B, beta coefficient; SE, standard error; CI, confidence interval
